# The Adapted POM Analysis of Avenanthramides In Silico

**DOI:** 10.3390/ph16050717

**Published:** 2023-05-09

**Authors:** Tibor Maliar, Mária Maliarová, Andrea Purdešová, Timotej Jankech, Ivana Gerhardtová, Patrik Beňovič, Václav Dvořáček, Michal Jágr, Jana Viskupičová

**Affiliations:** 1 Department of Chemistry, Faculty of Natural Sciences, University of Ss. Cyril and Methodius in Trnava, Námestie J. Herdu 2, 917 01 Trnava, Slovakia; maria.maliarova@ucm.sk (M.M.); andrea.purdesova@ucm.sk (A.P.); benovic2@ucm.sk (P.B.); 2 Institute of Neuroimmunology, Slovak Academy of Sciences, Dúbravská Cesta 9, 845 10 Bratislava, Slovakia; jankech7@uniba.sk (T.J.); gerhardtova1@uniba.sk (I.G.); 3 Department of Analytical Chemistry, Faculty of Natural Sciences, Comenius University in Bratislava, Mlynská Dolina, Ilkovičova 6, 842 15 Bratislava, Slovakia; 4 Crop Research Institute, Drnovská 507/73, 161 06 Prague, Czech Republic; dvoracek@vurv.cz (V.D.); jagr@vurv.cz (M.J.); 5 Centre of Experimental Medicine SAS, Institute of Experimental Pharmacology and Toxicology, Slovak Academy of Sciences, Dúbravská Cesta 9, 841 04 Bratislava, Slovakia; janaviskupicova@gmail.com

**Keywords:** avenanthramides, bioactivity, MOLINSPIRATION, OSIRIS, POM analysis, *Swiss-ADME*

## Abstract

POM analysis and related approaches are significant tools based on calculating various physico-chemical properties and predicting biological activity, ADME parameters, and toxicity of a molecule. These methods are used to evaluate a molecule’s potential to become a drug candidate. Avenanthramides (AVNs) are promising secondary metabolites specific to *Avena* spp. (oat). They comprise the amides of anthranilic acid linked to various polyphenolic acids with or without post-condensation molecule transformation. These natural compounds have been reported to exert numerous biological effects, including antioxidant, anti-inflammatory, hepatoprotective, antiatherogenic, and antiproliferative properties. To date, almost 50 various AVNs have been identified. We performed a modified POM analysis of 42 AVNs using MOLINSPIRATION, SWISSADME, and OSIRIS software. The evaluation of primary in silico parameters revealed significant differences among individual AVNs, highlighting the most promising candidates. These preliminary results may help coordinate and initiate other research projects focused on particular AVNs, especially those with predicted bioactivity, low toxicity, optimal ADME parameters, and promising perspectives.

## 1. Introduction

Avenanthramides (AVNs) are very promising secondary oat metabolites. They have been found predominantly in certain *Avena* species but also in minority sources such as white cabbage butterfly eggs (*Pieris brassicae* and *P. rapae*) [1] and in fungus-infected carnations (*Dianthus caryophyllus*) [2]. AVNs consist of conjugates of one of three phenylpropanoids (p-coumaric, ferulic, or caffeic acid) and anthranilic acid (or a hydroxylated and/or methoxylated derivative of anthranilic acid) [2]. Shortly summarized, AVNs are low molecular weight phenolic amides consisting of anthranilic acid linked to various polyphenolic acids with an amide bond with/without post-condensation modification. AVNs are considered particularly favorable plant secondary metabolites due to their ability to remain stable under conditions of higher temperature, slightly basic pH, and UV exposure [3].

Analytical papers about AVNs are mainly focused on characterizing the structural identity of AVNs. Currently, several tens of AVNs are known and described [4]. AVNs can be artificially synthesized. For example, AVNs A, B, D, and E were synthesized by Collins in 1989 using chromatography methods and by adapting the existing synthetic procedure of Bain and Smalley [5]. The most well-known synthetic AVN is Tranilast, which is an antiallergic drug marketed under the brand name Rizaben. It was developed by Kissei Pharmaceuticals and approved for use in Japan and South Korea for bronchial asthma in 1982. Indications for keloid and hypertrophic scars were added in the 1980s [6].

To date, a number of scientific studies have reported various biological effects of these compounds, particularly antioxidant [7], antiatherogenic [8], antiproliferative [9], anti-inflammatory [10], anti-itch/anti-irritant [11], and hepatoprotective [12] activities (reviewed in [13,14]). We have previously reported that certain types of AVNs were able to selectively inhibit proteinase activity, which is implicated in disease progression, especially in relation to pancreatitis, coagulation disorders, and cancer [15]. Moreover, recent in vivo studies and clinical trials have pointed out that AVNs or oat supplementation rich in AVNs may have a beneficial effect on cardiovascular disease [16,17], diseases associated with inflammation and oxidative stress [18], obesity, and lipid metabolism [19], as well as cancer [20].

PETRA/OSIRIS/MOLINSPIRATION (POM) analysis is an efficient bioinformatic approach used to assess the basic physical-chemical parameters of the molecules (drawn as structures) and predict bioactivity, toxicity, drug-likeness parameters, etc. The title of this approach consists of the software PETRA, OSIRIS, and the free online tool MOLINSPIRATION. PETRA (Parameter Estimation for the Treatment of Reactivity Applications) is a program package comprising various empirical methods for calculating basic physicochemical properties in organic molecules. All the methods are empirical in nature and have been developed over the last 20 years in the research group of Prof. J. Gasteiger. This software can quantify the following chemical effects: the heat of formation, bond dissociation energies, sigma charge distribution, π-charge distribution, inductive effect, resonance effect, delocalization energy, and polarizability effect [21]. The program OSIRIS (full name Osiris Property Explorer), originally developed by Thomas Sander, is a free tool for predicting physico-chemical (MW, clogP, water solubility, and total polar surface area—TPSA) and toxicological (mutagenic effect, tumorigenicity, irritancy, and reproductive toxicity) molecular properties. Based on these parameters, the program calculates drug-likeness properties and drug score [22]. MOLINSPIRATION offers a broad range of cheminformatics software tools supporting molecule manipulation and processing, including SMILES and SDfile conversion, normalization of molecules, generation of tautomers, molecule fragmentation, calculation of various molecular properties needed in QSAR, molecular modeling and drug design, high-quality molecule depiction, molecular database tools supporting substructure and similarity searches [23].

In this paper, the Petra software was replaced with the *SwissADME* tool because the prediction of pharmacological parameters was a priority. This free pharmacokinetic software allows the computation of physicochemical descriptors and prediction of ADME parameters, pharmacokinetic properties, drug-like nature, and medicinal chemistry friendliness of one or multiple molecules to support drug discovery and ensure safety [24]. There are several applications of this tool, and one of them is to produce two-dimensional models to identify and indicate the type of pharmacophore site that affects the biological activity with a change in the chemical substitution [25].

There is limited information available on the pharmacokinetic profile, potential toxicity, and metabolism of individual AVNs. Therefore, this study aims to provide a more in-depth analysis of these compounds, focusing on drug-likeness (drug score), toxicity, bioactivity, and favorable ADME properties. This study may be of particular interest to oat breeders and functional food manufacturers as well as a scientific community interested in the therapeutic potential of AVNs.

## 2. Results

The results of the MOLINSPIRATION calculation are provided in Table S1 in the Supplementary Material. AVNs are rather low molecular weight compounds (MW = 326.59–385.37 g/mol) and moderately polar compounds (logP = 0.75–3.55). The number of H-bond acceptors ranges from 4 to 9 for AVNs with one double bond, and from 5 to 8 for AVNs with two double bonds. Similarly, the number of H-bond donors ranges from 2 to 6 for AVNs with one double bond and from 3 to 6 for AVNs with two double bonds, while the number of rotatable bonds ranges from 4 to 7 for AVNs with one double bond and from 5 to 7 for AVNs with two double bonds. It is evident that AVNs are medium-large, mid-polar compounds with a higher number of H-bond acceptors than donors and a relatively high number of rotatable bonds. Based on the prediction score, AVNs appear to be promising bioactive compounds. Several AVNs with one double bond, such as the derivatives of 2-[(1-oxo-3-phenyl-2-propen-1-yl)amino]benzoic acid, showed a positive score as nuclear receptor ligands and enzyme inhibitors, which is an important structural feature for antineoplastic (oncolytic) effects. Similarly, several AVNs with two double bonds, i.e., the derivatives of 2-[[(2E,4E)-5-phenyl-1-oxo-2,4-pentadien-1-yl]amino]benzoic acid, exhibited a positive score for GPCR ligands. Based on the results summarized in Table S2, the following AVNs are particularly attractive, but only from the MOLINSPIRATION calculation point of view: 2c, 2ad, 2pd, or 5pd.

Table S2 shows the results of the OSIRIS calculation, which highlights differences among AVNs in terms of toxicity and drug-likeness. The analysis indicated a medium risk of reproductive toxicity or irritancy for the following AVNs: 1a, 1p, 1c, 1f, 1s, 2f, 2s, 2m, 3f, 3h(1), 3h(2), 5f, 5s, 1pd, 1cd, 1fd, and Tranilast. These mainly include derivatives of 2-[(1-oxo-3-phenyl-2-propen-1-yl)amino]benzoic acid. AVN 2ad was predicted to have a high risk of being an irritant. In general, AVNs are low-toxic and safe compounds, as supported by their low risk of tumorigenicity and mutagenicity. The values of clogP, TPSA, and MW are consistent with those obtained by MOLINSPIRATION. The color system’s advantage is that it provides quick orientation in the calculated results. Parameter TPSA critically influences drug-likeness or drug score, whereby the low polar surface area is desirable for a drug candidate. Based on TPSA, most of the AVNs, particularly (1a, 2a, 2p, 2c, 2f, 2s, 3p, 3c, 3f, 3h(1), 3h(2), 4p, 4c, 4f, 5a, 5p, 5c, 5f, 5s, AVN A2, 2pd, 2cd, 2fd, 2sd, 3pd, 3cd, 3fd, 5pd, 5cd, and 5fd) are out of the optimal range, which is related to symmetric derivatization of both aromatic rings on the molecule ends. Besides drug-likeness, the most important parameter is the drug score, which is calculated based on drug-likeness, cLogP, logS, molecular weight, and toxicity risks. From this point of view, the most favorable AVNs are 1s, 2p, 2c, 2m, 3c, 3h(2), 4p, 4c, 5p, 5c, 2pd, 2cd, 2fd, 2sd, 5cd, Tranilast, AVN A2, and dihydroavenanthramide D. Any relation to structural features, such as the exact position of OH and OCH_3_ functional groups on both aromatic rings, seems to be individual, and no general conclusion on the structure–activity relationship can be drawn.

The *SwissADME* calculation process examines AVNs from a pharmacokinetic perspective, including medicinal chemistry and drug-likeness score calculation parameters. The results are presented in Table S3. Almost all AVNs, except for 5c, 5s, and 5cd, showed good levels of gastrointestinal absorption. With the exception of AVN 1a, AVNs were predicted to be unable to cross the blood–brain barrier (BBB), likely due to the derivatization by OH and OCH_3_ functional groups. Furthermore, AVNs were predicted not to be substrates of P-glycoprotein (Pgp) and to serve as inhibitors of several cytochromes P450, indicating their potential influence on metabolization. Based on five different drug-likeness systems (Lipinski, Ghose, Weber, Egan, Muegge), AVNs are likely to be convenient drug candidates, except for 3c, 5c, 5f, 5s, 3fd, 5cd, and 5fd. Medicinal chemistry-related parameters, including the calculation of pan assays interference structure (PAINS), Brenk structural alert, lead-likeness, and synthetic accessibility score, indicate that 15 AVNs expressed “catechol alert” (PAINS), which may be related to the presence of two OH groups in the vicinal position. However, the majority of evaluated AVNs (except dihydroanthramide D) expressed 1-3 structural alerts (Brenk), such as catechol, michael_acceptor_1, and polyene, indicating less effective pharmacokinetics. Regarding skin permeation and bioavailability score values, the calculated results suggest that AVNs are expected to have good gastrointestinal absorption.

**Figure 1:**
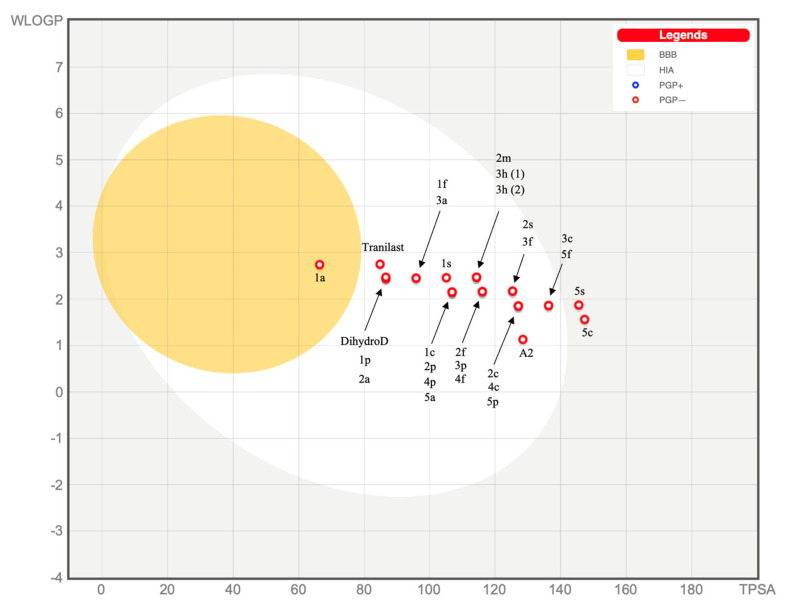

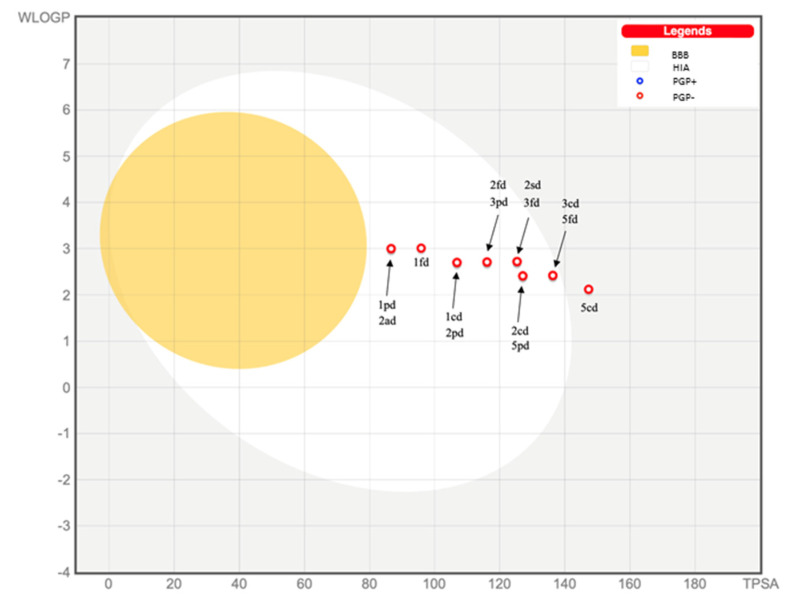
A “BOILED Egg” diagram for AVN derivatives of 2-[(1-Oxo-3-phenyl-2-propen-1-yl)amino]benzoic acid (upper chart) and 2-[[(2E,4E)-5-phenyl-1-oxo-2,4-pentadien-1-yl]amino]benzoic acid (lower chart).

The calculated synthetic accessibility score indicates that the entire group of AVNs is relatively easily synthesizable. Taken together, based on the *SwissADME* calculations, the following AVNs meet the drug-like criteria (GIA, BBB, no Pgp substrate, no CYP 450 inhibitors, positive (all five) drug-likeness parameters, no structural alerts, good skin permeation, and bioavailability score): AVN 1a, dihydroavenanthramide D and conditionally AVNs 1p, 1f, 1s, 4p, 4f, and Tranilast. The possibility to demonstrate the position of different AVNs in the “BOILED Egg” (Brain Or IntestinaL EstimateD permeation) diagram is particularly attractive. Compounds located in the white area fulfill the criteria for a drug candidate, meaning they can be passively absorbed by the gastrointestinal tract. If a compound appears at the same time in the yellow area, it can passively cross the BBB. Additionally, whether a compound is a Pgp substrate or not is indicated by color, with red dots indicating molecules predicted not to be affected by Pgp-mediated extrusion from the CNS. The BOILED Egg predictive model sets a threshold of WLOGP ≤ 5.88 and TPSA ≤ 131.6. The results are presented in Figure 1. It is evident that only AVN 1a is capable of crossing the blood–brain barrier (BBB). AVNs dihydro D, 1p, 2a, 1f, 3a, 1pd, 1fd, 1cd, and 2cd are located near the center of the white area, which indicates that they fulfill the requirements for drug candidates.

**Figure 2:**
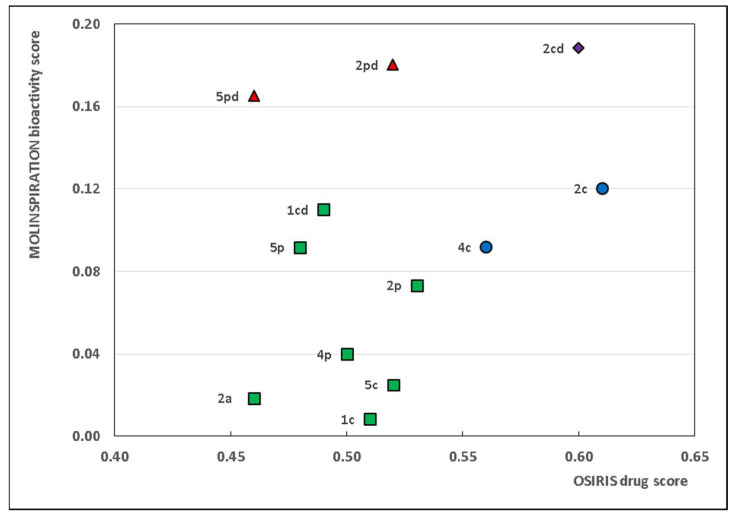
The sub-collection of AVNs with the best lead-likeness score, as calculated by *SwissADME*, including a MOLINSPIRATION final bioactivity score in intervals of positive values. The evaluated AVNs are marked as green squares, but the most promising substances are depicted as red triangles (+violet rhombus) and blue circles (+violet rhombus) based on MOLINSPIRATION bioactivity calculation and OSIRIS drug score prediction, respectively.

To obtain a comprehensive graphical representation of the most promising compounds based on their bioactivity score, optimal ADME, toxicity, and lead-likeness, we selected a sub-collection of AVNs with favorable calculated results. Initially, the collection of 42 AVNs was reduced to sub-collection 1 (*n* = 22) consisting of AVNs with a positive lead-likeness score based on *SwissADME* calculations, marked with ‘Y’ in Table S3. In the second step, a final MOLINSPIRATION bioactivity score was calculated by summing the maximal and average bioactivity scores. Sub-collection 1 was further reduced to sub-collection 2, consisting of AVNs with a positive value for the MOLINSPIRATION final bioactivity score, resulting in *n* = 12 compounds. Figure 2 displays a double plot of the 12 selected AVNs, presenting both the OSIRIS drug score and the MOLINSPIRATION final bioactivity score.

**Figure 3:**
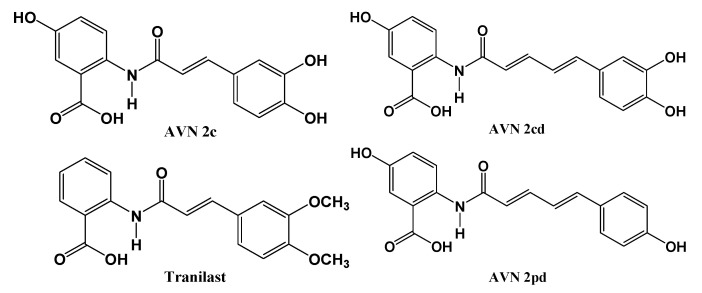
Structures of the most perspective AVNs.

Based on the results presented in Figure 2, only 12 out of the initial 42 AVNs met the requirement of having the best lead-likeness score (calculated by *SwissADME*) and a positive value of final bioactivity score (calculated by MOLINSPIRATION). According to the MOLINSPIRATION bioactivity and *SwissADME* lead-likeness score calculations, AVNs 5pd, 2pd, and 2cd appear to be the most promising compounds. However, based on the OSIRIS drug score and *SwissADME* lead-likeness score calculations, AVNs 4c, 2c, and 2cd are the most favorable candidates. The intersection of all three calculations contains AVN 2cd, which demonstrates an optimal lead-likeness score, no violations of drug-likeness, a relatively high bioactivity score, and favorable ADME properties with no observed toxicity. The structures of the most perspective compounds are presented in Figure 3, and the crucial calculated values of bioactivity and pharmacokinetics are given in Table 1.

The results presented in Table 1 show that the AVNs included have high GI absorption, good drug-likeness and drug score, and optimal lead-likeness, except for Tranilast, which has a lower MOLINSPIRATION bioactivity predicted score.

## 3. Discussion

Currently, there is no comprehensive SAR/POM analysis of avenanthramides available that considers their physico-chemical properties, bioactivities, ADMET, and pharmacokinetic profile. In the past, AVNs were referred to as avenalumins [26,27], and a structure–activity relationship analysis was published in 1994, focusing on isolated avenanthramide alkaloids and related compounds from the eggs of *Pieris brassicae* [1]. In one of the key databases, PUBCHEM, only the following AVNs have been referred to: 1c, 1f, 1p, 2c, 2f, 2p, 2s, A2, 4c, and 4p. Most published papers have focused on describing new avenanthramides using analytical methods, such as liquid chromatography-mass spectrometry (LC-MS) and these are mainly related to crop research. The publications are mostly related to the collection of AVNs rather than individual AVNs. While some AVNs mentioned are rare, most AVNs are published alongside AVN 2c. LC-MS analysis, based on the retention times of AVNs, indicates that these compounds have a mid-polar nature, which is characteristic of phenolic-like compounds [28,29]. Previous studies have suggested that reduced molecular flexibility, as measured by the number of rotatable bonds, and low polar surface area or total hydrogen bond count (sum of donors and acceptors) are important predictors of good oral bioavailability, regardless of molecular weight. It has been proposed that having 10 or fewer rotatable bonds and polar surface area equal to or less than 140 Å^2^ (or 12 or fewer H-bond donors and acceptors) increases the probability of good oral bioavailability [30]. All AVNs involved in this study meet the above-mentioned criteria. Additionally, it has been reported that estimating ADME (Absorption, Distribution, Metabolism, and Excretion) at an early stage in the discovery process can significantly reduce the fraction of pharmacokinetics-related failures during the clinical phases. Based on ADME parameters, these AVNs seem to be promising drug leads. Moreover, a drug candidate must exhibit strong biological efficacy while maintaining low toxicity. Our predicted results suggest that the AVNs included in the dataset are generally low toxic compounds, as supported by their low risk of tumorigenicity and mutagenicity.

The therapeutic potential of AVNs has not been clearly proven, as mentioned in the introduction. However, there is a growing body of scientific evidence suggesting that AVNs may have therapeutic potential, particularly in terms of their anti-inflammatory, antioxidant, and anticancer effects [31,32]. From the conjunction of three different calculations involved in our modified POM analysis, the AVN 2c, 2cd, and 2pd are perspective bioactive compounds besides synthetic standard Tranilast. From the data listed in Table 1, it is evident that all four AVNs exhibited high GI absorption, good drug-likeness, and drug score, and are considered good drug leads. Except for Tranilast, they also have a higher predicted bioactivity score by MOLINSPIRATION. Based on the achieved results, we could estimate the optimal pharmacophore, consisting of both options, AVNs with one or two double bonds, carrying appropriately localized two to three hydroxyl groups on both aromatic rings at positions R2, R3, and R4.

According to the SciFinder database, AVN 2c (CAS 116764-15-9) has been reported as a potent anti-inflammatory agent (108 records), exhibiting a moderating effect on various cytokines or inflammatory mediators such as interleukin 6, interleukin 1β, transcription factor NF-κB, and tumor necrosis factor, as well as a strong antioxidant (85 records) and a dietary supplement (25 records). In addition, AVN 2c acts as a suppressor of metalloproteinase-9 expression [33], suggesting its antiatherosclerosis activity. Other studies have shown that AVN 2c plays a role in protecting the intestine against ovalbumin-induced allergy via mediating the Hsp70-NF-κB signaling pathway [34], as well as its ability to protect against pyroptosis through ROS-induced mitochondrial damage via PI3K ubiquitination and phosphorylation in pediatric pneumonia [35], demonstrating its anti-inflammatory effect. Additionally, a cardioprotective effect has also been reported [36]. Similarly, avenanthramides 2cd (AVN L) and 2pd have been shown to exert a suppression of high-fat diet-induced atherosclerosis in Ldlr^-/-^ mice [16] and to exhibit anti-inflammatory activity [37]. All the biological effects of AVN 2c, 2cd, or 2pd mentioned are attributed to the favorable physico-chemical properties, optimal ADME parameters, and low (or selective) toxicity, thus confirming the predicted bioactivity calculated in this paper.

We must take into consideration that the amount of AVNs widely varies in oat grains. The literature reports the total concentration of AVAs in oats in the range from 2 to 289 mg/kg based on the cultivar, year, location, and cultivation conditions [38]. One interesting factor to consider is the stability during the processing of oats. In a study, the concentration of total AVAs (2c, 2p, and 2f) was reported to increase or to be restored in all oat products tested [3]. However, another study showed that the level of AVAs 2c and 2p decreased during commercial processing such as autoclaving and drum drying of oat grains [39]. Thus, the resulting health effect is a combination of several factors, such as the concentration of avenanthramides in oats, the types of AVNs contained in a particular cultivar, the absorption of a specific AVN in the small intestine, and the metabolism in the body.

## 4. Materials and Methods

### 4.1. POM Analysis

The software programs applied within POM analysis, particularly OSIRIS, MOLINSPIRATION, and *SwissADME*, operate on the same fundamental principle. Initially, the molecular structure is defined by either drawing it or inputting its SMILES (simplified molecular input line entry system) code. Subsequently, the specific parameters are calculated based on the fragment system.

### 4.2. MOLINSPIRATION Calculation

The MOLINSPIRATION calculation includes the Lipinski “drug-likeness” (Rule of five) parameters, which are as follows:

1. Octanol/water partition coefficient (LogP) is calculated as a sum of fragment-based contributions and correction factors.
2. Topological Polar Surface Area (TPSA) is calculated based on the methodology published by Ertl et al. [40] as a sum of fragment contributions. These have been obtained using the fitting sum of fragment contributions to “real” 3D volume for a training set of about twelve thousand, mostly drug-like molecules. Three-dimensional molecular geometries for a training set were fully optimized using the semiempirical AM1 method.
3. “Rule of 5” properties are a set of simple molecular descriptors used by Lipinski to formulate his “Rule of 5” [41]. The rule states, that most “drug-like” molecules have logP ≤ 5, molecular weight ≤ 500, number of hydrogen bond acceptors ≤ 10, and number of hydrogen bond donors ≤ 5. Molecules that violate more than one of these rules may have problems with bioavailability. The rule is called the “Rule of 5” because the border values are 5, 500, 2*5, and 5.
4. The number of Rotatable Bonds (nrotb) is a simple topological parameter that measures molecular flexibility. It has been shown to be a very good descriptor of the oral bioavailability of drugs [30]. A rotatable bond is defined as any single non-ring bond, bound to a non-terminal heavy (i.e., non-hydrogen) atom. Amide C-N bonds are not considered because of their high rotational energy barrier.

In the second step, the bioactivity score prediction using the MOLINSPIRATION virtual screening toolkit miscreen was calculated based on GPCR ligands (G-protein coupled receptors) /GPCRL/, ion channel modulators /ICHM/, kinase inhibitors /KI/, nuclear receptor ligands /NRL/, protease inhibitors /PI/, and other enzyme inhibitors /EI/.

### 4.3. OSIRIS Calculation

The OSRIS calculation parameters involved in the analysis are:

1. Toxicity endpoints (mutagenic, tumorigenic, irritant, and reproductive effective) are given by a “semaphore” light color.
2. The partition coefficient in n-octanol:water system (clogP) is calculated by incrementally adding contributions of each atom based on its atom type.
3. The solubility in water (logS) is calculated based on the stripped logarithm (base 10) of the solubility measured in mol/L.
4. Drug-likeness value prediction is based on calculating the equation that sums up the score values of fragments present in the molecule under investigation.
5. The drug score combines drug-likeness, cLogP, logS, molecular weight, and toxicity risks in one value that may be used to assess the compound’s overall potential to qualify as a drug.

### 4.4. SwissADME Calculation

The software [4] offers calculations in several sections, including:

1. Water solubility (logS, solubility class, calculated by three different systems).
2. Lipophilicity (logPo/w), calculated using five different approaches (ilogP, XlogP3, WlogP, MlogP, SILICOS-IT), and a consensus value (an average of the five calculations).
3. Basic physicochemical properties (molecular weight, number of heavy atoms, number of aromatic heavy atoms, fraction CSP3, number of rotatable bonds, number of H-bond acceptors, number of H-bond donors, molar refractivity and topological polar surface area /TPSA/).
4. Pharmacokinetic parameters, including gastrointestinal absorption /GI/, blood–brain barrier permeability /BBB/, inhibition of cytochrome P450 enzymes (1A2, 2C19, 2C9, 2D6, 3A4), and measure of skin permeation.
5. Medicinal chemistry calculations, such as pan assays interference structure /PAINS/, Brenk structural alert /BRENK/, lead-likeness /LL/, and synthetic accessibility score /SA/.
6. Drug-likeness score calculation using five different approaches (Lipinski, Ghose, Weber, Egan, Muegge) and bioavailability score.

For POM analysis, we selected pharmacokinetic, medicinal chemistry, and drug-likeness score parameters, as the remaining parameters showed similarity to those obtained from the OSIRIS and Molinspiration programs and were therefore excluded.

### 4.5. Structural Dataset

**Figure 4:**
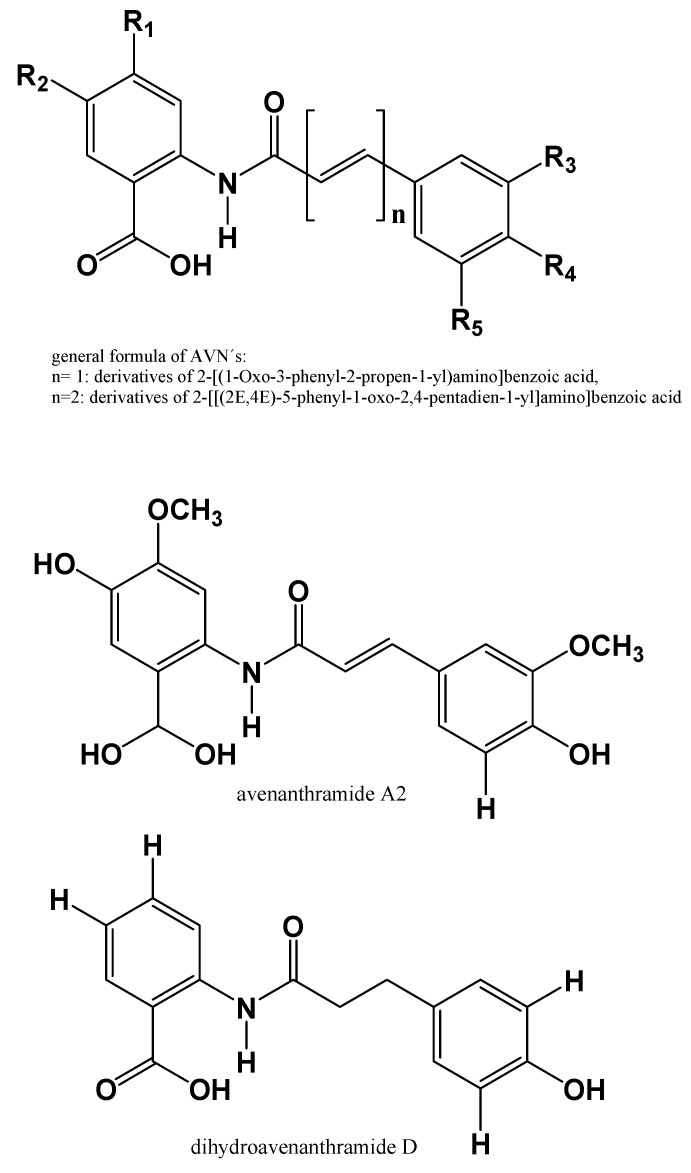
Structures of considered AVNs.

The individual structures of AVNs included in this study were obtained from various sources, including Heuschkel et al. [42], Kulichová et al. [40], Hernandez-Hernandez et al. [43], and Pubchem [44]. An additional 35 AVN structures were retrieved from Jágr et al. [6]. These AVNs were divided into two groups based on their derivation, either 2-[(1-Oxo-3-phenyl-2-propen-1-yl)amino]benzoic acid or 2-[[(2E,4E)-5-phenyl-1-oxo-2,4-pentadien-1-yl]amino]benzoic acid, as depicted in Figure 4.

## 5. Conclusions

Avenanthramides, a group of plant secondary metabolites found mainly in *Avena* spp., may offer potential as drug leads or food supplements due to their therapeutic properties. An increasing amount of scientific evidence indicates that AVNs might be particularly beneficial in terms of their anti-inflammatory, antioxidant, and anticancer properties. Modified POM analysis was performed to identify perspective AVN pharmacophore sites based on drug-likeness (score), bioactivity, toxicity, and ADME parameters. Our results suggest that AVNs are expected to be low-toxic and safe compounds with promising drug-like properties. Based on the POM analysis, we identified the most promising AVNs as 2c, 2cd, 2pd, and the standard Tranilast. The optimal pharmacophore was found to include AVNs that have one or two double bonds and two to three hydroxyl groups on both aromatic rings positioned at R2, R3, and R4. The information obtained may be useful in a particular sphere of interest, such as food, cosmetics, therapeutics, or crop breeding. However, follow-up research is needed to confirm the predicted results, i.e., the efficacy of Tranilast, 2c, 2cd, and 2pd and to determine their underlying molecular mechanisms and the implications for future applications. The continuous expansion of the avenanthramide dataset, including synthetic analogs, holds promise for future research. The application of POM analysis has shown promise in evaluating the biological activity of oat extract samples (cultivars) by assessing the partial contributions of individual AVNs. Further research in this area has the potential to lead to a better understanding of the health benefits of oats and the role of individual AVNs.

## Figures and Tables

**Table 1:** Bioactivity and pharmacokinetic properties for the most favorable AVNs and the standard Tranilast.

AVNs	MOLINSPIRATION Bioactivity Score			OSIRIS		*SwissADME*			
	MAX	AVG	MAX + AVG	Drug- Likeness	Drug Score	GI Absorption	BBB Permeant	Drug- Likeness	Lead-Likeness
2c	0.17	−0.05	0.12	−0.58	0.61	High	Yes	Yes	Yes
2cd	0.18	0.01	0.19	−0.38	0.6	High	No	Yes	Yes
2pd	0.18	0	0.18	−1.15	0.52	High	No	Yes	Yes
Tranilast	−0.02	−0.16	−0.18	2.74	0.51	High	No	Yes	Yes

## Data Availability

Data are contained within the article and Supplementary Material.

## Supplementary Materials

The following supporting information can be downloaded at: https://www.mdpi.com/article/10.3390/ph16050717/s1, Table S1: The Lipinski drug-likeness parameters of AVNs: highlighting positive bioactivity scores in yellow and optimal drug-like compounds in red. Table S2: The OSRIS calculations of AVNs provided in “semaphore” colors. Table S3: The *SwissADME* calculations of AVNs.
