# Supplementary material for: The Adapted POM Analysis of Avenanthramides In Silico
Source: Pharmaceuticals (Basel). 2023 May 9;16(5):717. doi: 10.3390/ph16050717 (PMC10224542; doi:10.3390/ph16050717)
Supplement: Supplementary file 1 [file pharmaceuticals-16-00717-s001.zip › pharmaceuticals-2383715-supplementary.pdf]

pharmaceuticals

MDPI

SUPPLEMENT

Table S1. The Lipinski drug-likeness parameters of AVNs: highlighting positive bioactivity scores in yellow and optimal drug-like compounds in red.

Labels Dinberg/ *Collins*	Subst.	milogP	TPSA (Å^{32})	nAtoms	MW (g/Mol)	nHBD	nHBA	nRB	nVIOL	Volume (Å^{3})	GPCRL	ICHM	KI	NRL	PI	EI
Derivatives of 2-[(1-Oxo-3-phenyl-2-propen-1-yl)amino]benzoic acid																
1a*	R_1-R_5=H	3.51	66.40	20	267.28	4	2	4	0	241.26	-0.18	-0.15	-0.29	-0.06	-0.31	-0.04
1p/*D*	R_4 =OH	3.03	86.62	21	283.28	5	3	4	0	249.27	-0.10	-0.10	-0.20	0.11	-0.27	0.01
1c/*F*	$R_{3,4}$ =OH	2.54	106.85	22	299.28	6	4	4	0	257.29	-0.09	-0.12	-0.20	0.10	-0.24	-0.00
1f/*E*	R_4 =OCH_3	2.85	95.86	23	313.31	6	3	5	0	274.82	-0.13	-0.20	-0.20	0.03	-0.29	-0.05
1s	$R_{3,5}$ =OH, R_4=OCH_3	2.87	105.09	25	343.33	7	3	6	0	300.37	-0.13	-0.18	-0.17	0.01	-0.23	-0.03
2a*	R_2 =OH	3.01	86.62	21	283.28	5	3	4	0	249.27	-0.11	-0.12	-0.22	0.12	-0.28	0.00
2p/*A*	$R_{2,4}$ =OH	2.53	106.85	22	299.28	6	4	4	0	257.29	-0.08	-0.11	-0.19	0.15	-0.23	0.00
2c/*C*	$R_{2,3,4}$ =OH	2.04	127.08	23	315.28	7	5	4	0	265.31	-0.05	-0.10	-0.15	0.17	-0.20	0.02
2f/*B*	$R_{2,4}$ =OH, R_3=OCH_3	2.35	116.09	24	329.31	7	4	5	0	282.84	-0.10	-0.17	-0.16	0.09	-0.26	-0.03
2s*	$R_{2,4}$ =OH, $R_{3,5}$=OCH_3	2.36	125.32	26	359.33	8	4	6	0	308.38	-0.12	-0.16	-0.14	0.06	-0.22	-0.01
2m*	R_2 =OH, $R_{3,4,5}$=OCH_3	2.64	114.33	27	373.36	8	3	7	0	325.91	-0.11	-0.17	-0.15	0.02	-0.23	-0.04
3a*	R_1 =OCH3, R_2 =OH,	2.83	95.86	23	313.31	6	3	5	0	274.82	-0.13	-0.19	-0.18	0.01	-0.30	-0.05
3p	R_1 =OCH3,	2.35	116.09	24	329.31	7	4	5	0	282.84	-0.10	-0.17	-0.15	0.07	-0.27	-0.04

	$R_{2,4}$ =OH,															
3c	R_1 =OCH3,<br>$R_{2,3,4}$ =OH,	1.86	136.31	25	345.31	8	5	5	0	290.86	-0.12	-0.18	-0.17	0.02	-0.25	-0.05
3f	$R_{1,3}$ =OCH3,<br>$R_{2,4}$ =OH,	2.17	125.32	26	359.33	8	4	6	0	308.38	-0.12	-0.18	-0.17	0.02	-0.24	-0.05
3h* (1)	$R_{1,3,5}$ =OCH3,<br>R_2 =OH,	2.87	114.33	27	373.36	8	3	7	0	325.91	-0.12	-0.18	-0.16	0.04	-0.24	-0.05
3h* (2)	$R_{1,3,4}$=OCH_3,<br>R_2 =OH,	2.47	114.33	27	373.36	8	3	7	0	325.91	-0.11	-0.17	-0.16	0.02	-0.23	-0.05
4p/G	$R_{1,4}$=OH,	2.53	106.85	22	299.28	6	4	4	0	257.29	-0.09	-0.10	-0.16	0.12	-0.23	0.00
4c/K	$R_{1,3,4}$=OH,	2.04	127.08	23	315.28	7	5	4	0	265.31	-0.06	-0.10	-0.14	0.15	-0.21	0.01
4f	$R_{1,,4}$=OH,<br>R_3 =OCH_3,	2.35	116.09	24	329.31	7	4	5	0	282.84	-0.10	-0.17	-0.15	0.07	-0.27	-0.04
5a*	$R_{1,2}$=OH,	2.52	106.85	22	299.28	6	4	4	0	257.29	-0.10	-0.12	-0.18	0.07	-0.25	-0.00
5p	$R_{1,3,4}$=OH,	2.04	127.08	23	315.28	7	5	4	0	265.31	-0.06	-0.10	-0.14	0.15	-0.21	0.01
5c	$R_{1,2,34}$=OH,	1.55	147.31	24	331.28	8	6	4	1	273.33	-0.07	-0.12	-0.16	0.10	-0.19	-0.01
5f	$R_{1,2,4}$=OH,<br>R_3=OCH_3,	1.86	136.31	25	345.31	8	5	5	0	290.86	-0.12	-0.18	-0.17	0.02	-0.25	-0.05
5s*	$R_{1,2,4}$=OH,<br>$R_{3,5}$=OCH_3,	1.87	145.55	27	375.33	9	5	6	0	316.40	-0.12	-0.16	-0.13	-0.00	-0.23	-0.01
Tranilast	$R_{3,4}$=OCH_3,	3.16	84.86	24	327.34	6	2	6	0	292.35	-0.14	-0.22	-0.22	-0.02	-0.25	-0.09
A2	$R_{1,3}$=OH,<br>$R_{2,4}$=OCH_3,	0.75	128.48	26	361.35	8	5	6	0	314.25	-0.09	-0.10	-0.09	0.03	-0.17	0.00
DHA D	R_4=OH,	3.00	86.62	21	285.30	5	3	5	0	255.46	-0.03	-0.07	-0.22	0.05	-0.13	-0.00
Derivatives of 2-[[(2E,4E)-5-phenyl-1-oxo-2,4-pentadien-1-yl]amino]benzoic acid																
1pd	R_4=OH,	3.55	86.62	23	309.32	5	3	5	0	276.69	0.02	-0.12	-0.03	0.17	-0.13	0.09
1cd	$R_{3,4}$=OH,	3.06	106.85	24	325.32	6	4	5	0	284.71	0.01	-0.14	-0.05	0.13	-0.13	0.07

1fd	R_3=OCH_3, R_4=OH,	3.37	95.86	25	339.35	6	3	6	0	302.24	-0.04	-0.20	-0.07	0.06	-0.20	0.02
2ad*	R_2=OH,	3.53	86.62	23	309.32	5	3	5	0	276.69	0.02	-0.13	-0.05	0.18	-0.14	0.08
2pd/L	$R_{2,4}$=OH,	3.05	106.85	24	325.32	6	4	5	0	284.71	0.02	-0.12	-0.04	0.18	-0.12	0.08
2cd	$R_{2,3,4}$=OH,	2.56	127.08	25	341.32	7	5	5	0	292.73	0.03	-0.11	-0.03	0.18	-0.11	0.09
2fd/P	$R_{2,4}$=OH, R_3=OCH_3,	2.87	116.09	26	355.35	7	4	6	0	310.25	-0.02	-0.18	-0.05	0.10	-0.18	0.04
2sd*	$R_{2,4}$=OH, $R_{3,5}$=OCH_3,	2.88	125.32	28	385.37	8	4	7	0	335.80	-0.04	-0.17	-0.04	0.07	-0.17	0.06
3pd	$R_{2,4}$=OH, R_1=OCH_3	2.87	116.09	26	355.35	7	4	6	0	310.25	-0.03	-0.18	-0.04	0.09	-0.20	0.04
3cd	$R_{2,3,4}$=OH, R_1=OCH_3	2.38	136.31	27	371.35	8	5	6	0	318.27	-0.04	-0.19	-0.06	0.04	-0.19	0.02
3fd	$R_{2,4}$=OH, $R_{1,3}$=OCH_3	2.68	125.32	28	385.37	8	4	7	0	335.80	-0.04	-0.18	-0.06	0.04	-0.19	0.02
5pd	$R_{1,2,4}$=OH	2.56	127.08	25	341.32	7	5	5	0	292.73	0.03	-0.11	-0.02	0.16	-0.12	0.09
5cd	$R_{1,2,3,4}$=OH	2.07	147.31	26	357.32	8	6	5	1	300.74	0.00	-0.13	-0.04	0.11	-0.12	0.07
5fd	$R_{1,2,4}$=OH, R_3=OCH_3	2.38	136.31	27	371.35	8	5	6	0	318.27	-0.04	-0.19	-0.06	0.04	-0.19	0.02

AVNs marked with * are only of synthetic origin.

milogP –partition coefficient in system n-ocatnol:water, TPSA – topological polar surface area, nAtoms – number of atomns, MW = molecular weight, nHBD-number of H-bond donors, nHBA-number of H-bond acceptors, nRB-number of rotatable bonds and bioactivity score prediction, calculated by MOLINSPIRATION

Table S2. The OSIRIS calculations of AVNs provided in "semaphore" colors.

**Labels Dinberg/ *Collins***	**Subst.**	**Mutagenic**	**Tumorigenic**	**Irritant**	**Reproductive effective**	**cLogP**	**Solubility**	**MW**	**TPSA**	**Druglikeness**	**Drug-Score**
Derivatives of 2-[(1-Oxo-3-phenyl-2-propen-1-yl)amino]benzoic acid											
1a*	R_1-R_5=H	•	•	•	•	2.62	-3.51	267.0	66.4	-0.92	0.35
1p/*D*	R_4 =OH	•	•	•	•	2.28	-3.21	283.0	86.63	0.62	0.46
1c/*F*	$R_{3,4}$ =OH	•	•	•	•	1.93	-2.92	299.0	106.8	1.46	0.51
1f/*E*	R_4 =OCH_3	•	•	•	•	2.21	-3.23	313.0	95.86	1.1	0.48
1s	$R_{3,5}$ =OH, R_4=OCH_3	•	•	•	•	2.14	-3.25	343.0	105.0	1.79	0.5
2a*	R_2 =OH	•	•	•	•	2.28	-3.21	283.0	86.63	-2.93	0.46
2p/*A*	$R_{2,4}$ =OH	•	•	•	•	1.93	-2.92	299.0	106.8	-1.37	0.53
2c/*C*	$R_{2,3,4}$ =OH	•	•	•	•	1.58	-2.62	315.0	127.0	-0.58	0.61
2f/*B*	$R_{2,4}$ =OH, R_3=OCH_3	•	•	•	•	1.86	-2.94	329.0	116.0	-0.94	0.45
2s*	$R_{2,4}$ =OH, $R_{3,5}$=OCH_3	•	•	•	•	1.79	-2.95	359.0	125.3	-0.15	0.5
2m*	R_2 =OH, $R_{3,4,5}$=OCH_3	•	•	•	•	2.07	-3.27	373.0	114.3	1.97	0.62
3a*	R_1 =OCH3, R_2 =OH,	•	•	•	•	2.21	-3.23	313.0	95.86	-3.33	0.44
3p	R_1 =OCH3, $R_{2,4}$ =OH,	•	•	•	•	1.86	-2.94	329.0	116.0	-1.8	0.5
3c	R_1 =OCH3, $R_{2,3,4}$ =OH,	•	•	•	•	1.51	-2.64	345.0	136.3	-0.97	0.56
3f	$R_{1,3}$ =OCH3, $R_{2,4}$ =OH,	•	•	•	•	1.79	-2.95	359.0	125.3	-1.45	0.41
3h* (1)	$R_{1,3,5}$ =OCH3,	•	•	•	•	2.07	-3.27	373.0	114.3	-11.08	0.33

	R_2 =OH,										
3h* (2)	$R_{1,3,4}$=OCH_3,<br>R_2 =OH,	●	●	●	●	2.07	-3.27	373.0	114.3	0.19	0.51
4p/G	$R_{1,4}$=OH,	●	●	●	●	1.93	-2.92	299.0	106.8	-1.93	0.5
4c/K	$R_{1,3,4}$=OH,	●	●	●	●	1.58	-2.62	315.0	127.0	-1.14	0.56
4f	$R_{1,,4}$=OH,<br>R_3 =OCH_3,	●	●	●	●	1.86	-2.94	329.0	116.0	-1.53	0.41
5a*	$R_{1,2}$=OH,	●	●	●	●	1.93	-2.92	299.0	106.8	-4.24	0.45
5p	$R_{1,3,4}$=OH,	●	●	●	●	1.58	-2.62	315.0	127.0	-2.66	0.48
5c	$R_{1,2,34}$=OH,	●	●	●	●	1.24	-2.33	331.0	147.3	-1.76	0.52
5f	$R_{1,2,4}$=OH,<br>R_3=OCH_3,	●	●	●	●	1.51	-2.64	345.0	136.3	-2.15	0.39
5s*	$R_{1,2,4}$=OH,<br>$R_{3,5}$=OCH_3,	●	●	●	●	1.44	-2.66	375.0	145.5	-1.42	0.41
Tranilast	$R_{3,4}$=OCH_3,	●	●	●	●	2.48	-3.55	327.0	84.86	2.74	0.51
A2	$R_{1,3}$=OH,<br>$R_{2,4}$=OCH_3,	●	●	●	●	1.6	-2.51	361.0	128.4	-0.23	0.63
DHA D	R_4=OH,	●	●	●	●	2.4	-3.08	285.0	86.63	1.28	0.78
Derivatives of 2-[[(2E,4E)-5-phenyl-1-oxo-2,4-pentadien-1-yl]amino]benzoic acid											
1pd	R_4=OH,	●	●	●	●	2.93	-3.53	309.0	86.63	0.83	0.44
1cd	$R_{3,4}$=OH,	●	●	●	●	2.59	-3.23	325.0	106.8	1.65	0.49
1fd	R_3=OCH_3,<br>R_4=OH,	●	●	●	●	2.86	-3.54	339.0	95.86	1.3	0.46
2ad*	R_2=OH,	●	●	●	●	2.93	-3.53	309.0	86.63	-6.42	0.25
2pd/L	$R_{2,4}$=OH,	●	●	●	●	2.59	-3.23	325.0	106.8	-1.15	0.52
2cd	$R_{2,3,4}$=OH,	●	●	●	●	2.24	-2.93	341.0	127.0	-0.38	0.6
2fd/P	$R_{2,4}$=OH,	●	●	●	●	2.52	-3.25	355.0	116.0	-0.72	0.55

	R_3=OCH_3,										
2sd*	$R_{2,4}$=OH, $R_{3,5}$=OCH_3,	●	●	●	●	2.45	-3.27	385.0	125.3	0.05	0.61
3pd	$R_{2,4}$=OH, R_1=OCH_3	●	●	●	●	2.52	-3.25	355.0	116.0	-1.6	0.48
3cd	$R_{2,3,4}$=OH, R_1=OCH_3	●	●	●	●	2.17	-2.95	371.0	136.3	-0.79	0.55
3fd	$R_{2,4}$=OH, $R_{1,3}$=OCH_3	●	●	●	●	2.45	-3.27	385.0	125.3	-1.25	0.49
5pd	$R_{1,2,4}$=OH	●	●	●	●	2.24	-2.93	341.0	127.0	-2.44	0.46
5cd	$R_{1,2,3,4}$=OH	●	●	●	●	1.9	-2.64	357.0	147.3	-1.56	0.51
5fd	$R_{1,2,4}$=OH, R_3=OCH_3	●	●	●	●	2.17	-2.95	371.0	136.3	-1.94	0.47

AVNs marked with * are of synthetic origin,

mutagenic, tumorigenic effect, irritant and reproductive effect, further clogP- calculated partition coefficient in system n-ocatnol:water, solubility in water, expressed as logS, MW- molecular weight, TPSA- topological polar surface area, drug-likeness, drug score is given as a circlet symbol, as "semaphore" color, (green color: non-toxic, values optimal; red color: toxic, non-optimal values, orange color: mild-toxic, mild optimal values) .

Table S3. The SwissADME calculations of AVNs.

Labels Dinberg/ *Collins*	Subst.	GIA	BBBP	P-gpS	CYP inhibitors	Lipinski	Ghose	Veber	Egan	Muegge	PAINS	Brenk	LL	SA	Log K_p	BAS
Derivatives of 2-[(1-Oxo-3-phenyl-2-propen-1-yl)amino]benzoic acid																
1a*	R_1-R_5=H	H	Y	N	N,N,Y,N,N	Y	Y	Y	Y	Y	-		XLOGP3 > 3.5	2.26	-5.12	0.85
1p/*D*	R_4 =OH	H	N	N	N,N,Y,N,N	Y	Y	Y	Y	Y	-		XLOGP3 > 3.5	2.24	-5.46	0.56
1c/*F*	$R_{3,4}$ =OH	H	N	N	N,N,N,N,N	Y	Y	Y	Y	Y	catechol_A	Michael_acceptor_1	Y	2.35	-6.32	0.56
1f/*E*	R_4 =OCH_3	H	N	N	N,N,Y,N,N	Y	Y	Y	Y	Y	-		Y	2.46	-6.17	0.56
1s	$R_{3,5}$ =OH, R_4=OCH_3	H	N	N	N,N,Y,N,N	Y	Y	Y	Y	Y	-		Y	2.66	-6.37	0.56
2a*	R_2 =OH	H	N	N	N,N,N,N,N	Y	Y	Y	Y	Y	-	hydroquinone, Michael_acceptor_1	Y	2.32	-5.96	0.56
2p/*A*	$R_{2,4}$ =OH	H	N	N	N,N,N,N,N	Y	Y	Y	Y	Y	-		Y	2.36	-6.32	0.56
2c/*C*	$R_{2,3,4}$ =OH	H	N	N	N,N,N,N,N	Y	Y	Y	Y	Y	catechol_A	catechol, hydroquinone, Michael_acceptor_1	Y	2.47	-6.75	0.56
2f/*B*	$R_{2,4}$ =OH, R_3=OCH_3	H	N	N	N,N,Y,N,N	Y	Y	Y	Y	Y	-		Y	2.57	-6.52	0.56
2s*	$R_{2,4}$ =OH, $R_{3,5}$=OCH_3	H	N	N	N,N,Y,N,N	Y	Y	Y	Y	Y	-		MW > 350	2.77	-6.72	0.56
2m*	R_2 =OH, $R_{3,4,5}$=OCH_3	H	N	N	N,N,Y,N,N	Y	Y	Y	Y	Y	-	hydroquinone, Michael_acceptor_1	MW > 350, Rotors > 7	2.89	-6.58	0.56
3a*	R_1 =OCH3, R_2 =OH,	H	N	N	N,N,Y,N,N	Y	Y	Y	Y	Y	-		Y	2.60	-6.17	0.56
3p	R_1 =OCH3, $R_{2,4}$ =OH,	H	N	N	N,N,Y,N,N	Y	Y	Y	Y	Y	-		Y	2.64	-6.52	0.56
3c	R_1 =OCH3, $R_{2,3,4}$ =OH,	H	N	N	Y,N,N,N,N	Y	Y	Y	N	Y	catechol_A	catechol, hydroquinone, Mi-	Y	2.73	-6.95	0.56

												chael_acceptor_1				
3f	$R_{1,3}$ =OCH3, $R_{2,4}$ =OH,	H	N	N	N,N,Y,N,N	Y	Y	Y	Y	Y	-	hydroquinone, Michael_acceptor_1	MW > 350	2.85	-6.72	0.56
3h* (1)	$R_{1,3,5}$ =OCH3, R_2 =OH,	H	N	N	N,N,Y,N,N	Y	Y	Y	Y	Y	-	hydroquinone,	MW > 350, Rotors > 7	3.01	-6.58	0.56
3h* (2)	$R_{1,3,4}$=OCH_3, R_2 =OH,	H	N	N	N,N,Y,N,N	Y	Y	Y	Y	Y	-	Michael_acceptor_1	MW > 350, Rotors > 7	2.96	-6.58	0.56
4p/G	$R_{1,4}$=OH,	H	N	N	N,N,N,N,N	Y	Y	Y	Y	Y	-	Michael_acceptor_1	Y	2.41	-6.32	0.56
4c/K	$R_{1,3,4}$=OH,	H	N	N	N,N,N,N,N	Y	Y	Y	Y	Y	catechol_A	catechol, Michael_acceptor_1	Y	2.51	-6.75	0.56
4f	$R_{1,,4}$=OH, R_3 =OCH_3,	H	N	N	N,N,Y,N,N	Y	Y	Y	Y	Y	-	Michael_acceptor_1	Y	2.61	-6.52	0.56
5a*	$R_{1,2}$=OH,	H	N	N	N,N,N,N,N	Y	Y	Y	Y	Y	catechol_A	catechol, hydroquinone, Michael_acceptor_1	Y	2.50	-6.32	0.56
5p	$R_{1,3,4}$=OH,	H	N	N	N,N,N,N,N	Y	Y	Y	Y	Y	catechol_A		Y	2.53	-6.67	0.56
5c	$R_{1,2,34}$=OH,	L	N	N	Y,N,N,N,N	nHBD > 5	Y	TPSA > 140	TPSA > 131.6	nHBD> 5	catechol_A		Y	2.62	-7.01	0.56
5f	$R_{1,2,4}$=OH, R_3=OCH_3,	H	N	N	Y,N,Y,N,N	Y	Y	Y	TPSA > 131.6	Y	catechol_A	catechol, hydroquinone, Michael_acceptor_1	Y	2.72	-6.87	0.56
5s*	$R_{1,2,4}$=OH, $R_{3,5}$=OCH_3,	L	N	N	Y,N,Y,N,N	Y	Y	TPSA > 140	TPSA > 131.6	Y	catechol_A		MW > 350	2.91	-7.07	0.56
Tranilast	$R_{3,4}$=OCH_3,	H	N	N	N,N,Y,N,N	Y	Y	Y	Y	Y	-	Michael_acceptor_1	Y	2.57	-6.02	0.56
A2	$R_{1,3}$=OH, $R_{2,4}$=OCH_3,	H	N	N	Y,N,Y,N,N	Y	Y	Y	Y	Y	-	het-C-het_not_in_ring, hydroquinone,	MW > 350	2.89	-7.50	0.55

												Michael_acceptor_1				
DHA D	R_4=OH,	H	N	N	N,N,N,N,N	Y	Y	Y	Y	Y	-	-	Y	1.80	-5.62	0.56
Derivatives of 2-[[(2E,4E)-5-phenyl-1-oxo-2,4-pentadien-1-yl]amino]benzoic acid																
1pd	R_4=OH,	H	N	N	N,N,Y,N,N	Y	Y	Y	Y	Y	-	Michael_acceptor_1, polyene	No; 1 violation: XLOGP > 3.5	2.64	-5.17	0.56
1cd	$R_{3,4}$=OH,	H	N	N	N,N,Y,N,N	Y	Y	Y	Y	Y	catechol_A	catechol, Michael_acceptor_1, polyene	Y	2.73	-6.02	0.56
1fd	R_3=OCH_3, R_4=OH,	H	N	N	N,N,Y,N,N	Y	Y	Y	Y	Y	-	Michael_acceptor_1, polyene	XLOGP > 3.5	2.82	-5.87	0.56
2ad*	R_2=OH,	H	N	N	N,N,Y,N,N	Y	Y	Y	Y	Y	-	hydroquinone,	XLOGP > 3.5	2.70	-5.67	0.56
2pd/L	$R_{2,4}$=OH,	H	N	N	N,N,Y,N,N	Y	Y	Y	Y	Y	-	Michael_acceptor_1, polyene	Y	2.74	-6.02	0.56
2cd	$R_{2,3,4}$=OH,	H	N	N	N,N,Y,N,N	Y	Y	Y	Y	Y	catechol_A	catechol, hydroquinone, Michael_acceptor_1, polyene	Y	2.83	-6.37	0.56
2fd/P	$R_{2,4}$=OH, R_3=OCH_3,	H	N	N	N,N,Y,N,N	Y	Y	Y	Y	Y	-		MW > 350	2.92	-6.22	0.56
2sd*	$R_{2,4}$=OH, $R_{3,5}$=OCH_3,	H	N	N	N,N,Y,N,N	Y	Y	Y	Y	Y	-	hydroquinone, Michael_acceptor_1, polyene	MW > 350, Rotors > 7	3.11	-6.42	0.56
3pd	$R_{2,4}$=OH, R_1=OCH_3	H	N	N	N,N,Y,N,N	Y	Y	Y	Y	Y	-		MW > 350	2.98	-6.22	0.56
3cd	$R_{2,3,4}$=OH, R_1=OCH_3	H	N	N	N,N,Y,N,N	Y	Y	Y	TPSA > 131.6	Y	catechol_A	catechol, hydroquinone, Michael_acceptor_1, polyene	MW > 350		-6.57	0.56

3fd	$R_{2,4}$=OH, $R_{1,3}$=OCH_3	H	N	N	N,N,Y,N,N	Y	Y	Y	Y	Y	-	hydroquinone, Michael_acceptor_1, polyene	MW > 350, Rotors > 7	-6.42	0.56
5pd	$R_{1,2,4}$=OH	H	N	N	N,N,Y,N,N	Y	Y	Y	Y	Y	catechol_A		Y	-6.37	0.56
5cd	$R_{1,2,3,4}$=OH	L	N	N	Y,N,Y,N,N	Y	Y	TPSA > 140	TPSA > 131.6	: H-don > 5	catechol_A	catechol, hydroqui-none, Mi-chael_acceptor_1, polyene	MW > 350	-6.72	0.56
5fd	$R_{1,2,4}$=OH, R_3=OCH_3	H	N	N	N,N,Y,N,N	Y	Y	Y	TPSA > 131.6	Y	catechol_A		MW > 350	-6.57	0.56

AVNs marked with * are of synthetic origin,

Y-Yes, N – No, H = High,

GIA - gastrointestinal absorption, BBB = Blood brain barrier permeability, P-gpS – P-glycoprotein substrate, CYP inhibitors - cytochrome P450 (in exact order 1A2, 2C19, 2C9, 2D6, 3A4) inhibitors, drug-likeness by five different approaches /Lipinski, Ghose, Veber, Egan, Muegge/, PAINS - pan assay interference structures, Brenk - structural alert by Brenk, LL – lead-likeness, SA - synthetic accessibility, BAS - bioavailability score /BAS/.
